# Supplementary material for: A Peripheral Blood Signature of Increased Th1 and Myeloid Cells Combined with Serum Inflammatory Mediators Is Associated with Response to Abatacept in Rheumatoid Arthritis Patients
Source: Cells. 2023 Dec 9;12(24):2808. doi: 10.3390/cells12242808 (PMC10741898; doi:10.3390/cells12242808)
Supplement: Supplementary file 1 [file cells-12-02808-s001.zip › cells-2709616-supplementary.pdf]

Figure S1

Sensitivity analysis of the composite index as a predictor of clinical responses

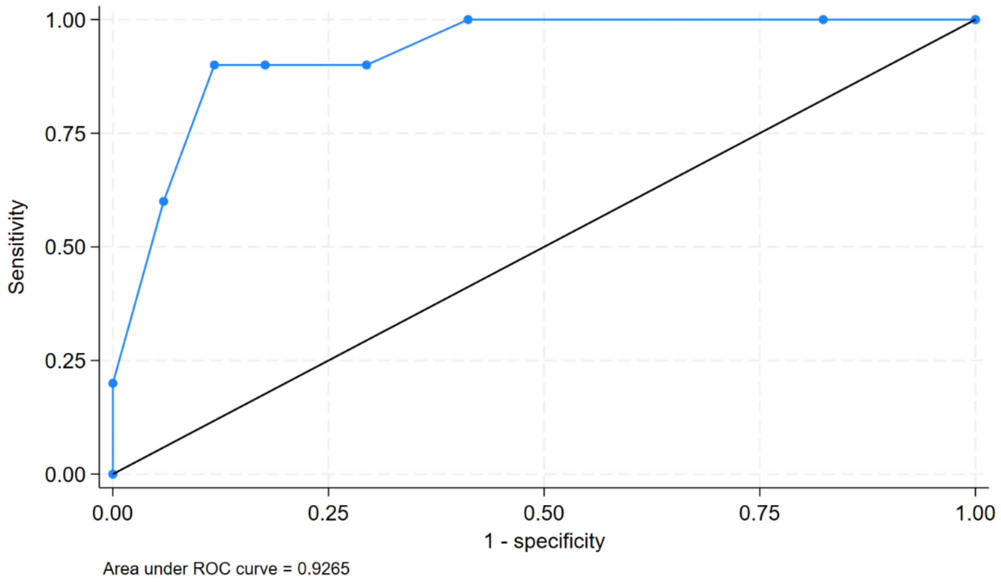

Detailed report of sensitivity and specificity

Cutpoint	Sensitivity	Specificity	Correctly classified	LR+	LR-
(>= 0)	100.00%	0.00%	37.04%	1.0000	
(>= 1)	100.00%	17.65%	48.15%	1.2143	0.0000
(>= 2)	100.00%	58.82%	74.07%	2.4286	0.0000
(>= 3)	90.00%	70.59%	77.78%	3.0600	0.1417
(>= 4)	90.00%	82.35%	85.19%	5.1000	0.1214
(>= 5)	90.00%	88.24%	88.89%	7.6500	0.1133
(>= 6)	60.00%	94.12%	81.48%	10.2000	0.4250
(>= 8)	20.00%	100.00%	70.37%		0.8000
(> 8)	0.00%	100.00%	62.96%		1.0000

Obs	ROC area	Std. err.	Asymptotic normal [95% conf. interval]	
27	0.9265	0.0492	0.82995	1.00000
